# Opponent Hitting Behavior Prediction and Ball Location Control for a Table Tennis Robot

**DOI:** 10.3390/biomimetics8020229

**Published:** 2023-05-29

**Authors:** Yunfeng Ji, Yue Mao, Fangfei Suo, Xiaoyi Hu, Yunfeng Hou, Ye Yuan

**Affiliations:** 1 Institute of Machine Intelligence, University of Shanghai for Science and Technology, Shanghai 200093, China; 2 School of Health Science and Engineering, University of Shanghai for Science and Technology, Shanghai 200093, China

**Keywords:** table tennis robot, visual module, hitting behavior prediction, ball location control

## Abstract

The hitting position and velocity control for table tennis robots have been investigated widely in the literature. However, most of the studies conducted do not consider the opponent’s hitting behaviors, which may reduce hitting accuracy. This paper proposes a new table tennis robot framework that returns the ball based on the opponent’s hitting behaviors. Specifically, we classify the opponent’s hitting behaviors into four categories: forehand attacking, forehand rubbing, backhand attacking, and backhand rubbing. A tailor-made mechanical structure that consists of a robot arm and a two-dimensional slide rail is developed such that the robot can reach large workspaces. Additionally, a visual module is incorporated to enable the robot to capture opponent motion sequences. Based on the opponent’s hitting behaviors and the predicted ball trajectory, smooth and stable control of the robot’s hitting motion can be obtained by applying quintic polynomial trajectory planning. Moreover, a motion control strategy is devised for the robot to return the ball to the desired location. Extensive experimental results are presented to demonstrate the effectiveness of the proposed strategy.

## 1. Introduction

Recent advances in science and technology have generated higher requirements for robot mobility, adaptability and survival ability [1,2,3]. To satisfy these requirements, robots are required to sense changing conditions occurring in the environment so that appropriate control decisions can be made [4,5,6,7,8,9]. Recently, a variety of bionic robots have been developed to complete various tasks, such as high-speed flexible movement [10], jumping [11], swimming [12], walking [13], etc. However, how to effectively react to changing environments remains a challenging task. For example, in table tennis robot systems, how can we accurately predict the trajectory of a spinning ball? One common approach is to measure the rotations of the ball via a high-speed camera by setting a mark on it [14,15]. However, these methods are conservative since they put a high requirement on the performances of the camera and the measuring algorithms. It is common sense that the players predict the rotation types of the ball according to the opponent’s hitting behaviors. Inspired by this, this paper develops techniques to map the opponent’s hitting behaviors to the rotation types of the ball.

Robot-arm-based table tennis robots have been studied for more than 40 years [16,17]. The authors of [18] developed a table tennis robot system that consists of a 7-degrees-of-freedom (DoF) industrial robot arm DARM-2 and two linear cameras. This robot has successfully completed two to three rounds against the wall. By using a 6-DoF PUMA260 industrial robot arm and four high-speed cameras, the table tennis robot [19] has achieved man-machine matchmaking for the first time. With the rapid development of hardware and software technology, the successful rate of returning the ball for the table tennis robot [20] has attained 58%. In [21], the table tennis robot can play with humans for up to 50 rounds. Additionally, the authors of [22] developed a self-designed lightweight robot arm, which can achieve quick and flexible strokes. In [23], a humanoid table tennis robot was developed. The robot was designed according to the human skeleton and was able to achieve improved human-machine interaction.

Notwithstanding these advances, most of the mentioned robots have the following issues: (1) Fixed robotic arms and humanoid table tennis robots can only hit the ball within a limited workspace; (2) There is no control over the attitude of the racket; (3) Current studies ignore the perception and prediction of the opponent’s hitting behavior.

To deal with these issues, we develop a bionic table tennis robot in this paper that can reach large workspaces and return the ball to the desired location based on the opponent’s hitting behaviors. The developed robot shows high adaptability and stability. Specifically, we first develop the vision system of the table tennis robot for tracking the trajectory of the ball and capturing the opponent’s action. Based on the opponent’s behavior, procedures are developed to predict the rotation type of the ball as well as the trajectory of the ball. To return the ball precisely, a dynamic model of the robot arm is established. Then, based on the quintic polynomial trajectory planning algorithm, we realize dynamic trajectory planning even when the desired endpoint of the robot arm is changing constantly. Finally, we show how to return the ball to the desired location. Note that, in our previous work [24], we discussed how to predict the trajectory of the ball and return the ball to the desired location. This work extends our previous work by considering the opponent’s stroke behaviors when returning the ball. Compared with [24], the approach developed in this paper has a higher success rate of returning the ball to the target area. In addition, benefiting from the stroke behavior prediction, the developed robot system can respond to changes in the environment more quickly compared with [24].

The rest of this paper is organized as follows: Section 2 introduces the overall structure of our table tennis robot. Section 3 introduces the visual system. Section 4 develops the robot arm control algorithm. Section 5 elaborates on the location control of the ball. Section 6 describes the experiments undertaken and provides analysis of the results. Section 7 summarizes the paper and presents proposals for our future work.

## 2. The Table Tennis Robot System

The developed table tennis robot system consists of a vision module, an execution module, and a control module.

**Figure 1:**
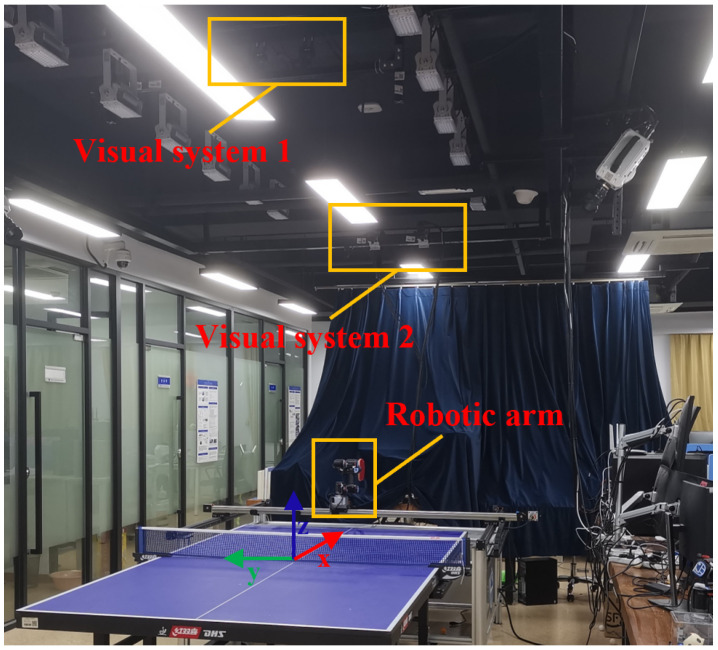
The developed table tennis robot system.

As shown in Figure 1, the vision module is composed of two visual systems, denoted by visual systems 1 and 2, respectively. Each visual system contains two Baumer HXC20 high-speed monochromatic industrial cameras. Cameras are installed above the table tennis table to track the trajectory of the ping-pong ball for visual system 1. Cameras are installed above the robot arm to track the stroke motion of the player for visual system 2. Two slide rails with a length of 185 cm are installed outside the two long sides of the table, and another slide rail, with a length of 220 cm, is placed on the two slides on both sides, enabling horizontal movement of the robot with 2-DoF. The control module is used for visual processing, data communication, and manipulator motion control.

The world coordinate system of the overall system is defined as follows: the origin of the system is located at the center of the table, the positive *x*-axis points to the robot along the centerline on the table, the positive *y*-axis points to the right side of the robotic arm along the width of the table, and the positive *z*-axis is vertical and upward.

## 3. The Vision Module

### 3.1. Trajectory Prediction of Balls

Let us first discuss how to track the trajectory of the ball online. Specifically, we develop a moving object detection algorithm, which combines techniques of background subtraction and color segmentation. We first obtain the color threshold of the ball by comparing different images under different light conditions. It is shown that the color threshold of the ball is [176,255]. Then, for each image, we recognize the ball by executing background subtraction, image binarization, morphology open operation, and corrosion expansion operation sequentially. Additionally, to improve the recognition accuracy and reduce the recognition time, we adopt the region of interest (ROI) algorithm to narrow the scan area of each image.

The internal and external parameters of the camera are obtained by Zhang’s calibration method [25]. We conduct the coordinate transformation using the perspective-n-point (PnP) algorithm [26]. Then, we can obtain the three-dimensional coordinates of the ball.

To control the robot to play table tennis with humans, it is crucial to predetermine the possible hitting point so that the robot can return the ball to the desired location in real-time. To achieve this, we need to predict the trajectory of the ball, which was solved in our previous work [24]. Roughly speaking, the process consists of three steps. First, a dynamic model of the ball is derived; second, to obtain the initial velocity of the ball, we develop an initial velocity correction method based on the feedback of the location of the ball; third, based on the dynamic model of the ball and the computed initial velocity, we calculate the hitting position of the ball. We refer the reader to [24] for more details.

### 3.2. Stroke Type Classification and Rotation Type Prediction

**Figure 2:**
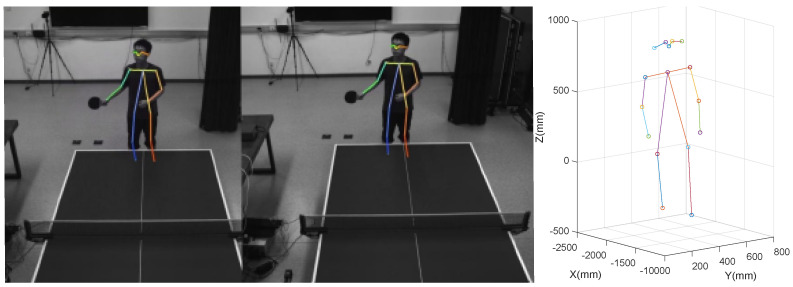
Three-dimensional coordinates of the key points.

In this paper, we adopt the Fast Pose 17 human posture model [27]. The 17 key points of the human body for this model are given in Table 1. As shown in Figure 2, the binocular camera first identifies the location of the key points, and then obtains the 3D position of each key point via the 3D reconstruction. The identification error of the coordinate position of the key points is within 2 cm, which satisfies the experimental requirements.

**Figure 3:**
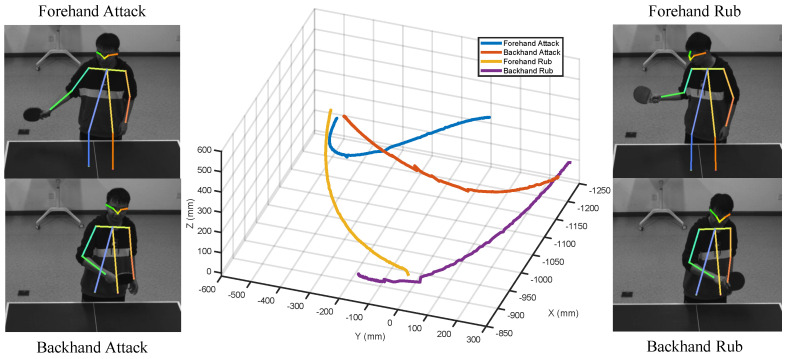
Four types of strokes.

As shown in Table 2, each type of ball rotation corresponds to a different type of stroke, which includes forehand attacking, forehand rubbing, backhand attacking, and backhand rubbing. To better return a ball, we need to identify the stroke type of the opponent. To this end, for each stroke type, we collect 500 motion sequences of coordinate of the opponent’s racket hand, as shown in Figure 3. Based on the collected data, the SVM (support vector machine) can be trained and then used to predict the stroke type of the opponent.

Since the stroke types of the opponent and the motion sequence of the wrist are highly correlated, we select the position of the wrist as the feature point of the SVM algorithm. Specifically, we use the Fast Pose 17 human posture model to locate the position of a human’s wrist. Then, the current velocity of the wrist is predicted by the Kalman filter algorithm [28]. Each feature vector of the SVM consists of velocities of the wrist in the past 20 samplings. For each stroke type, different control strategies to the end attitude of the robot arm are performed. The details of the control are discussed in the following sections.

**Figure 4:**
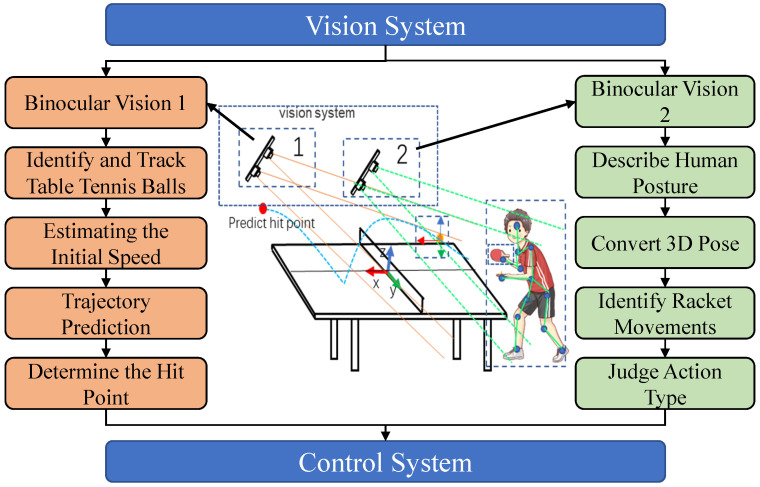
Flowchart of the visual system.

By predicting the opponent’s hitting behavior and trajectory, we can simulate the flight path and the rotation direction of the ball. The overall working diagram for the visual module is depicted in Figure 4.

## 4. Humanoid Robotic Arm Control

**Figure 5:**
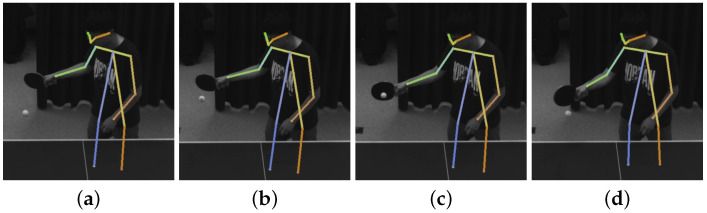

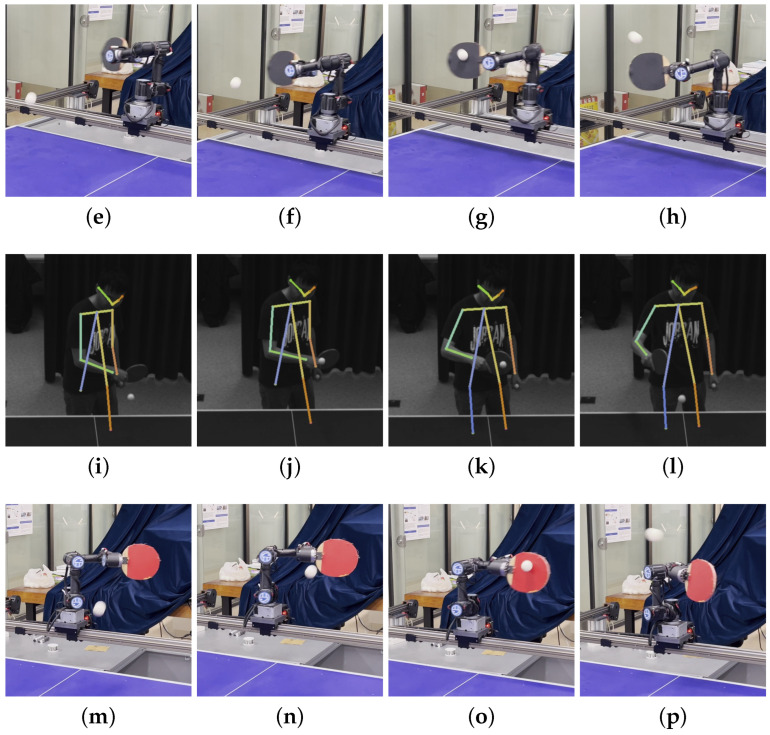
Forehand and backhand stroke simulations. (**a**) Forehand stroke: stage 1; (**b**) Forehand stroke: stage 2; (**c**) Forehand stroke: stage 3; (**d**) Forehand stroke: stage 4; (**e**) Forehand stroke imitation: stage 1; (**f**) Forehand stroke imitation: stage 2; (**g**) Forehand stroke imitation: stage 3; (**h**) Forehand stroke imitation: stage 4; (**i**) Backhand stroke: stage 1; (**j**) Backhand stroke: stage 2; (**k**) Backhand stroke: stage 3; (**l**) Backhand stroke: stage 4; (**m**) Backhand stroke imitation: stage 1; (**n**) Backhand stroke imitation: stage 2; (**o**) Backhand stroke imitation: stage 3; (**p**) Backhand stroke imitation: stage 4.

To return the ball, we design a humanoid 4-DoF robotic arm. Figure 5 shows how the humanoid robotic arm simulates the forehand and backhand attacking or rubbing of a player. Specifically, Figure 5a–d show that the player has completed a forehand stroke; in Figure 5e–h, the robot imitates the forehand stroke behaviors shown in Figure 5a–d, respectively. Similarly, Figure 5i–l illustrate that the player has completed a backhand stroke, and in Figure 5m–p, the robot imitates the backhand stroke behaviors shown in Figure 5i–l, respectively.

**Figure 6:**
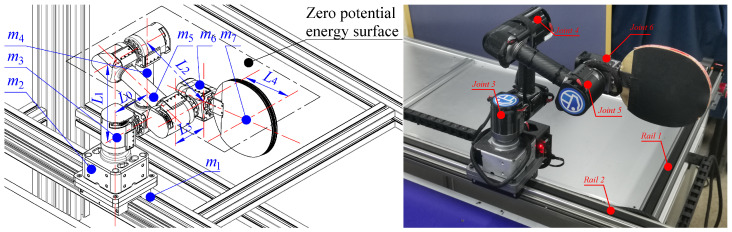
The structure of the robot arm.

As shown in Figure 6, the execution module of the robot is comprised of the 4-DoF robotic arm and the two-dimensional slide rail. The robot is anthropomorphic in the sense that: (i) a 2-DOF rail (Rails 1 and 2) is used to imitate the movement of the human; (ii) joints 3 and 4 are used to imitate the rotations of the human waist and elbow, respectively; (iii) joints 5 and 6 function as a universal joint to imitate the human wrist. Benefiting from the anthropomorphic design of the robot arm, it can improve the accuracy of returning the ball.

To track the designed acceleration and velocity of the robot in real time, we need to analyze the robot’s dynamic model. To this end, we first analyze the kinematics of the robot arm. As shown in Figure 6, we establish the coordinate system of the robot. The D-H parameter method is used to obtain the position and attitude of the racket. The D-H parameters of the table tennis robot system can be found in our previous work [24].

### 4.1. Joint Position Calculation

Here, *i* denotes the *i*th joint, $d_{i}$ denotes the offset at the joint *i* axis, $\theta_{i}$ is the angle of joint *i*, and $L_{1},\ldots ,L_{4}$ are illustrated in Figure 6. The homogeneous transformation matrix between two adjacent coordinate systems can be derived as ${}_{i}^{i-1}T$. The transformation matrix from the base to the terminal of the robot arm can be calculated as follows:

(1) $$\begin{array}{cc} \; & {}_{6}^{0}T={}_{1}^{0}T{}_{2}^{1}T{}_{3}^{2}T{}_{4}^{3}T{}_{5}^{4}T{}_{6}^{5}T=\left[\begin{array}{cc} {}_{6}^{0}R & {}_{6}^{0}P\\ 0 & 1 \end{array}\right] \end{array}$$

(2) $$\begin{array}{cc} \; & {}_{6}^{0}R=\left[\begin{array}{ccc} {}_{6}^{0}R_{1} & {}_{6}^{0}R_{2} & {}_{6}^{0}R_{3} \end{array}\right] \end{array}$$

(3) $$\begin{array}{cc} \; & {}_{6}^{0}R_{1}=\left[\begin{array}{c} c\left(\theta_{4}\right)c\left(\theta_{5}\right)s\left(\theta_{6}\right)s\left(\theta_{3}\right)-c\left(\theta_{3}\right)s\left(\theta_{6}\right)-c\left(\theta_{6}\right)s\left(\theta_{3}\right)s\left(\theta_{4}\right)s\left(\theta_{5}\right)\\ c\left(\theta_{3}\right)c\left(\theta_{6}\right)s\left(\theta_{4}\right)s\left(\theta_{5}\right)-c\left(\theta_{3}\right)c\left(\theta_{4}\right)c\left(\theta_{5}\right)c\left(\theta_{6}\right)-s\left(\theta_{3}\right)s\left(\theta_{6}\right)\\ s\left(\theta_{4}+\theta_{5}\right)c\left(\theta_{6}\right) \end{array}\right] \end{array}$$

(4) $$\begin{array}{cc} \; & {}_{6}^{0}R_{2}=\left[\begin{array}{c} s\left(\theta_{3}\right)s\left(\theta_{4}\right)s\left(\theta_{5}\right)s\left(\theta_{6}\right)-c\left(\theta_{4}\right)c\left(\theta_{5}\right)s\left(\theta_{3}\right)s\left(\theta_{6}\right)-c\left(\theta_{3}\right)c\left(\theta_{6}\right)\\ c\left(\theta_{3}\right)c\left(\theta_{4}\right)c\left(\theta_{5}\right)s\left(\theta_{6}\right)-c\left(\theta_{6}\right)s\left(\theta_{3}\right)-c\left(\theta_{3}\right)s\left(\theta_{4}\right)s\left(\theta_{5}\right)s\left(\theta_{6}\right)\\ -s\left(\theta_{4}+\theta_{5}\right)s\left(\theta_{6}\right) \end{array}\right] \end{array}$$

(5) $$\begin{array}{cc} \; & {}_{6}^{0}R_{3}=\left[\begin{array}{c} s\left(\theta_{4}+\theta_{5}\right)s\left(\theta_{3}\right)\\ -s\left(\theta_{4}+\theta_{5}\right)c\left(\theta_{3}\right)\\ -c(\theta_{4}+\theta_{5}) \end{array}\right] \end{array}$$

(6) $$\begin{array}{cc} \; & {}_{6}^{0}P=\left[\begin{array}{c} d_{2}-L_{2}c\left(\theta_{3}\right)+L_{3}c\left(\theta_{4}\right)s\left(\theta_{3}\right)+L_{4}c\left(\theta_{4}\right)s\left(\theta_{3}\right)s\left(\theta_{5}\right)+L_{4}c\left(\theta_{5}\right)s\left(\theta_{3}\right)s\left(\theta_{4}\right)\\ -d_{1}-L_{2}s\left(\theta_{3}\right)-L_{3}c\left(\theta_{3}\right)c\left(\theta_{4}\right)-L_{4}c\left(\theta_{3}\right)c\left(\theta_{4}\right)s\left(\theta_{5}\right)-L_{4}c\left(\theta_{3}\right)c\left(\theta_{5}\right)s\left(\theta_{4}\right)\\ L_{1}-L_{4}-c\left(\theta_{4}+\theta_{5}\right)+L_{3}s\left(\theta_{4}\right) \end{array}\right] \end{array}$$

where ${}_{6}^{0}R$ and ${}_{6}^{0}P$ represent the rotation matrix and the translation matrix between the base coordinate system and the terminal coordinate system, respectively; c(θ) represents cos(θ); s(θ) represents sin(θ). To precisely control the posture and position of the racket, an algebraic approach is applied to obtain the inverse kinematic model. The elements are transformed from Euler angles or quaternions as:

(7) $$\begin{array}{c} {}_{6}^{0}T_{m}=\left[\begin{array}{cccc} n_{x} & o_{x} & a_{x} & p_{x}\\ n_{y} & o_{y} & a_{y} & p_{y}\\ n_{z} & o_{z} & a_{z} & p_{z}\\ 0 & 0 & 0 & 1 \end{array}\right] \end{array}$$

Based on the D-H parameters, we obtain all the required variables by substituting into the transformation matrix ${}_{i}^{i-1}T$.

By Algorithm 1, there are 16 possible solutions of the robot inverse kinematics, of which 2–8 solutions are correct, and the remaining solutions are wrong. In practical applications, it is necessary to estimate the correctness of the solutions in real-time and then choose the most appropriate solution according to the optimal control principle of the shortest displacement.


**Algorithm 1:** Solving parameters of ${}_{6}^{0}T_{m}$
Solving $\theta_{4}+\theta_{5}=\pm a\cos\left(a_{z}\right)$
Solving $\theta_{6}=\pm a\cos\left(n_{z}/\sin\left(\theta_{4}+\theta_{5}\right)\right)$
Solving $\theta_{3}=\pm a\cos\left(n_{y}\cos\left(\theta_{6}\right)-o_{y}\sin\left(\theta_{6}\right)/a_{z}\right)$
Solving $\theta_{4}=\pm a\sin\left(\left(p_{z}-L_{4}a_{z}-L_{1}\right)/L_{3}\right)$ or
(8)$$\begin{array}{c} \theta_{4}=\left\{\begin{array}{cc} \pi -a\sin(\left(p_{z}-L_{4}a_{z}-L_{1}\right)/L_{3}) & \mathrm{if}\;0\leq a\sin(\left(p_{z}-L_{4}a_{z}-L_{1}\right)/L_{3})\leq \pi /2\\ -\pi -a\sin(\left(p_{z}-L_{4}a_{z}-L_{1}\right)/L_{3}) & \mathrm{if}\;-\pi /2\leq a\sin(\left(p_{z}-L_{4}a_{z}-L_{1}\right)/L_{3})\leq 0 \end{array}\right. \end{array}$$
Solving $\theta_{5}=\left(\theta_{4}+\theta_{5}\right)-\theta_{4}$
Solving $d_{1}=-p_{y}-L_{2}s\left(\theta_{3}\right)-L_{3}c\left(\theta_{3}\right)c\left(\theta_{4}\right)-L_{4}c\left(\theta_{3}\right)c\left(\theta_{4}\right)s\left(\theta_{5}\right)-L_{4}c\left(\theta_{3}\right)c\left(\theta_{5}\right)s\left(\theta_{4}\right)$
Solving $d_{2}=p_{x}+L_{2}c\left(\theta_{3}\right)-L_{3}c\left(\theta_{4}\right)s\left(\theta_{3}\right)-L_{4}c\left(\theta_{4}\right)s\left(\theta_{3}\right)s\left(\theta_{5}\right)-L_{4}c\left(\theta_{5}\right)s\left(\theta_{3}\right)s\left(\theta_{4}\right)$



### 4.2. Joint Velocity Calculation and Trajectory Planning

To return the ball successfully, we need to control the robot to reach the final hitting point quickly and accurately while preserving the continuous acceleration and jerk of the motors and avoiding vibrations and shocks on the joints. To this end, we execute the trajectory planning for all joints of the robot arm to ensure its accuracy and stability and to keep it as continuous as possible.

First, we need to obtain the angular velocity and the angle of each joint when the racket reaches the final hitting point and meets the expected hitting velocity. The angle of rotation of each joint can be obtained by inverse kinematics, and the angular velocity of each joint can be obtained by

(9) $$\begin{array}{c} {\left[\begin{array}{cccccc} v_{d1} & v_{d2} & v_{\theta 3} & v_{\theta 4} & v_{\theta 5} & v_{\theta 6} \end{array}\right]}^{T}=J^{-1}{\left[\begin{array}{cccccc} v_{x} & v_{y} & v_{z} & \omega_{x} & \omega_{y} & \omega_{z} \end{array}\right]}^{T} \end{array}$$

where $v_{d1}$ and $v_{d2}$ are the linear velocities of the two slide rails, $v_{\theta 3}∼v_{\theta 6}$ are the angular velocities of the joints of the robot arm, $v_{x}$, $v_{y}$, $v_{z}$ are the linear velocities of the racket in the *x*, *y*, and *z* directions, respectively, $\omega_{x}$, $\omega_{y}$, $\omega_{z}$ are the angular velocities of the racket in the *x*, *y*, and *z* directions, respectively, and $J^{-1}$ is the inverse matrix of the Jacobi matrix of the table tennis robot.

Next, we perform trajectory planning for each joint of the robot using quintic polynomials as follows.

(10) $$\begin{array}{c} \theta_{i}\left(t\right)=c_{i0}+c_{i1}t+c_{i2}t^{2}+c_{i3}t^{3}+c_{i4}t^{4}+c_{i5}t^{5} \end{array}$$

Substituting the computed $c_{i0}∼c_{i5}$ into (10), we obtain the position that the racket will reach in the next moment $t_{s}$. The above process is repeated after each $t_{s}$. Note that $\theta_{i}\left(t_{s}\right)$ and ${\dot{\theta }}_{i}\left(t_{s}\right)$ are given by the stroke decision returned by the vision module and are reset after each stroke. In addition, note that ${\ddot{\theta }}_{i}\left(t_{s}\right)=0$, i.e., the angular acceleration of joint *i* is zero when hitting the ball. In this way, we can drive the racket to the desired position with the desired velocity. The trajectory planning for this stroke is complete.

## 5. Ball Location Control

In this section, we first establish the collision rebound model. A square area of 60 cm in length and width is set as the target area to return the ball. Then, based on the established collision rebound model, we can calculate the stroke speed and angle for the robot to return the ball to the target area. When the ball collides with the table, its velocity will decrease in both the horizontal and vertical directions. The collision rebound model can be expressed by:

(11) $$\begin{array}{c} \vec{V_{out}}=K_{t}\vec{V_{in}}+\vec{B_{t}}, \end{array}$$

where $K_{t}$ is a diagonal matrix representing the velocity loss coefficient after the ball collides with the table, $\vec{B_{t}}$ is the velocity compensation bias after the collision, $\vec{V_{in}}$ is the incident velocity of the collision between the ball and the table, and $\vec{V_{out}}$ is the exit velocity after the collision between the ball and the table. The coordinates of $\vec{V_{in}}$ and $\vec{V_{out}}$ should be relatively static with respect to the collision plane.

The axes of these coordinates are perpendicular to the collision plane and point in the ball’s incoming direction.

The flight trajectory of the ball after the collision can be obtained through the prediction model according to the calculated exit velocity $\vec{V_{out}}$ and the coordinates of the collision point.

Before each stroke, the flight trajectory of the incoming ball can be predicted, and the hitting position, velocity, and time can also be calculated. To return the ball to the desired location, we can compute the initial velocity of the ball based on feedback on the location of the ball. Specifically, according to the hitting position $P_{0}$ and the central point of the target area $P_{n}$, we can obtain the desired velocity of the ball after hitting. By using the collision rebound model, the transformation matrix of the ball’s velocity between the world coordinate system and the racket coordinate system can be derived as follows:

(12) $$\begin{array}{c} R=\left[\begin{array}{ccc} -\cos(\phi )\sin(\psi ) & \cos(\phi )\cos(\psi ) & \sin(\phi )\\ -\sin(\psi )\sin(\phi ) & \cos(\psi )\sin(\phi ) & -\cos(\phi )\\ -\cos(\psi ) & -\sin(\psi ) & 0 \end{array}\right] \end{array}$$

where ϕ and ψ are the pitch and psi in the Tait–Bryan angles.

In addition to the collision rebound model between the ball and the table, there is a similar collision rebound model between the ball and the racket. We have

(13) $$\begin{array}{c} R\left(\vec{V_{r}^{w}}-\vec{V_{O}}\right)=K_{r}R\left(\vec{V_{I}}-\vec{V_{O}}\right)+\vec{B_{r}}, \end{array}$$

where $\vec{V_{I}}=\left[v_{Ix},v_{Iy},v_{Iz}\right]$, $\vec{V_{O}}=\left[v_{Ox},v_{Oy},v_{Oz}\right]$, and $\vec{V_{r}^{w}}=\left[v_{rx},v_{ry},v_{rz}\right]$ are the velocity of the ball before the collision, the velocity of the ball after the collision, and the velocity of the racket with respect to the world coordinate system. Similarly, $K_{r}$ is a diagonal matrix representing the velocity loss coefficient between the ball and the racket, $k_{x}$, $k_{y}$, $k_{z}$ are the elements on the diagonal of the $K_{r}$, and $\vec{B_{r}}$ is the velocity compensation bias after the collision. Let the velocities of the ball in the directions of *Z* and *Y* be 0. Then, we can obtain the following nonlinear equation system:

(14) $$\begin{array}{cc} \; & v_{Oz}S_{\phi }-b_{x}-v_{Iz}k_{x}S_{\phi }+v_{Oy}C_{\phi }C_{\psi }+C_{\phi }S_{\psi }\left(v_{rx}^{w}-v_{Ox}\right)-v_{Iy}k_{x}C_{\phi }C_{\psi }-k_{x}C_{\phi }S_{\psi }\left(v_{rx}^{w}-v_{Ix}\right)=0 \end{array}$$

(15) $$\begin{array}{cc} \; & v_{Oy}C_{\psi }S_{\phi }-v_{Oz}C_{\phi }-b_{y}+S_{\phi }S_{\psi }\left(v_{rx}-v_{Ox}\right)+v_{Iz}k_{y}C_{\phi }-k_{y}S_{\phi }S_{\psi }\left(v_{rx}-v_{Ix}\right)-v_{Iy}k_{y}C_{\psi }S_{\phi }=0 \end{array}$$

(16) $$\begin{array}{cc} \; & C_{\psi }\left(v_{rx}-v_{Ox}\right)-v_{Oy}S_{\psi }-b_{z}+v_{Iy}k_{z}S_{\psi }-k_{z}C_{\psi }\left(v_{rx}-v_{Ix}\right)=0, \end{array}$$

where $S_{\phi }=\sin\left(\phi \right)$, $C_{\phi }=\cos\left(\phi \right)$, $S_{\psi }=\sin\left(\psi \right)$, and $C_{\psi }=\cos\left(\psi \right)$.

By solving these equations, we can obtain the racket speed and attitude for returning the ball to the desired area. Because of the complexity of the nonlinear equations, it is difficult to directly calculate the analytical solution. To deal with this issue, we propose Algorithm 2 to approach the solutions from the initial value by using the Newton method.

The reason we use a linear model to model the ping-pong ball collision is that we consider the collision to be inelastic. Therefore, the problem of modeling the collision of ping-pong balls can be solved by determining the $K_{t}$, $K_{r}$, $\vec{B_{t}}$ and $\vec{B_{r}}$. By trial and error, we get

(17) $$K_{t}=\left[\begin{array}{ccc} 0.50259 & 0 & 0\\ 0 & 0.75 & 0\\ 0 & 0 & -0.9 \end{array}\right]\vec{B_{t}}=\left[\begin{array}{c} 0.50652\\ 0\\ 0 \end{array}\right]\;\;K_{r}=\left[\begin{array}{ccc} 0.82 & 0 & 0\\ 0 & -0.88 & 0\\ 0 & 0 & -0.82 \end{array}\right]\vec{B_{r}}=\left[\begin{array}{c} 0.1\\ 0\\ 0.1 \end{array}\right]$$

**Algorithm 2:** Solutions of the nonlinear equations
*Step 1*: Let Equation (14) be $f_{1}$, Equation (15) be $f_{2}$, and Equation (16) be $f_{3}$.
Let $\phi_{0}=\pi /2$, $v_{rx0}=-2m/s$, $\psi_{0}=\pi /2$ if Y>0, and $\psi_{0}=-\pi /2$ if Y≤0.
Let the jacobian matrix $F^{\prime }$ be
$$\begin{array}{c} F^{\prime }\left(\phi ,\psi ,v_{rx}\right)=\left[\begin{array}{ccc} \frac{\partial f_{1}}{\partial \phi } & \frac{\partial f_{1}}{\partial \psi } & \frac{\partial f_{1}}{\partial v_{rx}}\\ \frac{\partial f_{2}}{\partial \phi } & \frac{\partial f_{2}}{\partial \phi } & \frac{\partial f_{2}}{\partial v_{rx}}\\ \frac{\partial f_{3}}{\partial \phi } & \frac{\partial f_{3}}{\partial \psi } & \frac{\partial f_{3}}{\partial v_{rx}} \end{array}\right] \end{array}$$
*Step 2*: Solving elements of $F^{\prime }$: $\frac{\partial f_{1}}{\partial \phi }$, $\frac{\partial f_{1}}{\partial \psi }$, $\frac{\partial f_{1}}{\partial v_{rx}}$, $\frac{\partial f_{2}}{\partial \phi }$, $\frac{\partial f_{2}}{\partial \psi }$, $\frac{\partial f_{2}}{\partial v_{rx}}$, $\frac{\partial f_{3}}{\partial \phi }$, $\frac{\partial f_{3}}{\partial \psi }$, $\frac{\partial f_{3}}{\partial v_{rx}}$
*Step 3*: Let $S_{0}=\left[\begin{array}{c} \phi_{0}\\ \psi_{0}\\ v_{rx0} \end{array}\right]$.
*Step 4*: Calculate $S_{1}=S_{0}-F^{\prime }{\left(S_{0}\right)}^{-1}\left[\begin{array}{c} f_{1}\left(S_{0}\right)\\ f_{2}\left(S_{0}\right)\\ f_{3}\left(S_{0}\right) \end{array}\right]$.
*Step 5*: If $S_{1}-S_{0}\leq 10^{-6}$, return $S_{1}$, and otherwise, set $S_{0}\leftarrow S_{1}$ and go to Step 4.



Using the inverse kinematics, the desired joint position can be calculated. Then, by applying the trajectory planning in the joint space, the angle, angular velocity, and angular acceleration of each joint of the robot can be uniquely determined over time.

## 6. Experiment

**Figure 7:**
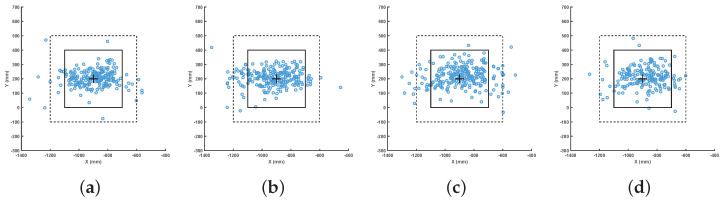
Actual landing points and target area (4 experiments with same target point). (**a**) Landing points of the returned ball: forehand attack; (**b**) Landing points of the returned ball: backhand attack; (**c**) Landing points of the returned ball: forehand rub; (**d**) Landing points of the returned ball: backhand rub.

**Figure 8:**
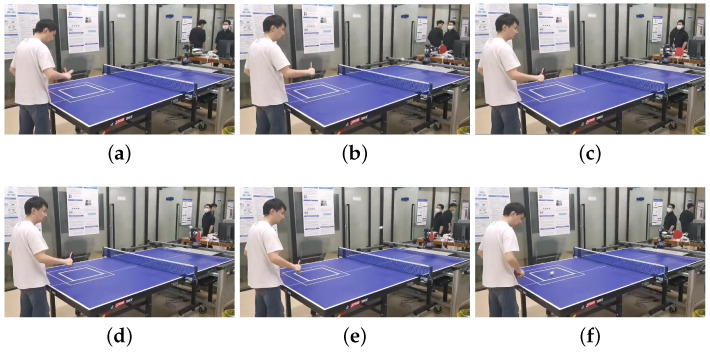
Experimental process. (**a**) Backhand attack: touching; (**b**) Backhand attack: flying; (**c**) Backhand attack: landing; (**d**) Backhand attack return: touching; (**e**) Backhand attack return: flying; (**f**) Backhand attack return: landing.

As shown in Figure 7 and Figure 8, a target square area is prespecified and (−900,200) is set as the target point. In Figure 7, the squares composed of the dotted lines and solid lines correspond to the outer square and the inner square, respectively, in Figure 8. Moreover, to clearly show the performance of our method, we use “+” to mark the target point.

In this experiment, the robot plays against a player who returns the ball at a random velocity and angle. Specifically, the robot plays a total of 20,648 rounds to approximate the performance of the robot precisely. The stroke type prediction accuracy and success rate of returning the ball to the target area are shown in Table 3 and Table 4. In Table 3, the prediction accuracy of the model for forehand attack, backhand attack, forehand rub and backhand rub is 95.52%, 94.8%, 93.17% and 93.41% respectively. It can be seen that the rub stroke has a worse prediction accuracy. This is mostly because our model has a higher propensity to identify the rub as an attack. The detail of the experimental results are shown in Figure 7.

Following further investigation, we discovered that misidentification was the primary cause of several balls failing to strike the inner target area. Our identification system may mistake the player’s white cuff for a ball, thus supplying the robot with the incorrect position of the ball. Although the influence of this error can be gradually eliminated in trajectory prediction, if the recognition error is too large, the robot’s performance will suffer and the ball will finally travel out of bounds.

## 7. Conclusions

This paper proposed a bionic table tennis robot system. A novel mechanical structure and a robust algorithm were designed. Extensive experimental results demonstrated that the algorithm can effectively improve the stability and accuracy of the robot’s performance. However, due to the limitations of the mechanical structure, the robot failed in most of the cases where the returned ball had a high speed exceeding 10 m/s as well as strong rotation. In the future, both the structure and algorithm of the table tennis robot will be further refined to enable the robot to return the ball with even greater speed and spin.

## Figures and Tables

**Table 1:** Key points of the Fast Pose 17 model.

Number	Key Point	Number	Key Point
0	Nose	9	Left wrist
1	Left eye	10	Right wrist
2	Right eye	11	Left waist
3	Left ear	12	Right waist
4	Right ear	13	Left knee
5	Left shoulder	14	Right knee
6	Right shoulder	15	Left ankle
7	Left elbow	16	Right ankle
8	Right elbow		

**Table 2:** Strokes and the corresponding types of the rotation.

Type	Stroke	Rotation
1	Forehand attacking	Topspin, left sidespin
2	Backhand attacking	Topspin, right sidespin
3	Forehand rubbing	Backspin, left sidespin
4	Backhand rubbing	Backspin, right sidespin

**Table 3:** Stroke type prediction accuracy.

Stroke Type	Accuracy
Forehand attack	95.52%
Backhand attack	94.8 %
Forehand rub	93.17%
Backhand rub	93.41%

**Table 4:** Success rate of returning the ball to the target area.

Rounds	Hitting Success Rate	Within the Inner Target Area	Within the Outler Target Area	Mean Placement of the Ball
20,648	98.35%	82.52%	95.16%	(−875.13, 178.27)

## Data Availability

The data presented in this study are available on request from the corresponding author. The data are not publicly available due to privacy restrictions.
